# A comprehensive meta-analysis of tissue resident memory T cells and their roles in shaping immune microenvironment and patient prognosis in non-small cell lung cancer

**DOI:** 10.3389/fimmu.2024.1416751

**Published:** 2024-07-08

**Authors:** Aidan Shen, Aliesha Garrett, Cheng-Chi Chao, Dongliang Liu, Chao Cheng, Zhaohui Wang, Chen Qian, Yangzhi Zhu, Junhua Mai, Chongming Jiang

**Affiliations:** ^1^ Department of Precision Medicine, Terasaki Institute for Biomedical Innovation, Los Angeles, CA, United States; ^2^ Department of Pipeline Development, Biomap, Inc., San Francisco, CA, United States; ^3^ Michael E. DeBakey Department of Surgery, Baylor College of Medicine, Houston, TX, United States; ^4^ Department of Medicine, Baylor College of Medicine, Houston, TX, United States; ^5^ Department of Medicine, Keck School of Medicine, University of Southern California, Los Angeles, CA, United States; ^6^ Department of Nanomedicine, Houston Methodist Research Institute, Houston, TX, United States

**Keywords:** tissue resident memory T cell, non-small-cell lung cancer, prognosis, tumor immune microenvironment, machine learning

## Abstract

Tissue-resident memory T cells (T_RM_) are a specialized subset of long-lived memory T cells that reside in peripheral tissues. However, the impact of T_RM_-related immunosurveillance on the tumor-immune microenvironment (TIME) and tumor progression across various non-small-cell lung cancer (NSCLC) patient populations is yet to be elucidated. Our comprehensive analysis of multiple independent single-cell and bulk RNA-seq datasets of patient NSCLC samples generated reliable, unique T_RM_ signatures, through which we inferred the abundance of T_RM_ in NSCLC. We discovered that T_RM_ abundance is consistently positively correlated with CD4+ T helper 1 cells, M1 macrophages, and resting dendritic cells in the TIME. In addition, T_RM_ signatures are strongly associated with immune checkpoint and stimulatory genes and the prognosis of NSCLC patients. A T_RM_-based machine learning model to predict patient survival was validated and an 18-gene risk score was further developed to effectively stratify patients into low-risk and high-risk categories, wherein patients with high-risk scores had significantly lower overall survival than patients with low-risk. The prognostic value of the risk score was independently validated by the Cancer Genome Atlas Program (TCGA) dataset and multiple independent NSCLC patient datasets. Notably, low-risk NSCLC patients with higher T_RM_ infiltration exhibited enhanced T-cell immunity, nature killer cell activation, and other TIME immune responses related pathways, indicating a more active immune profile benefitting from immunotherapy. However, the T_RM_ signature revealed low T_RM_ abundance and a lack of prognostic association among lung squamous cell carcinoma patients in contrast to adenocarcinoma, indicating that the two NSCLC subtypes are driven by distinct TIMEs. Altogether, this study provides valuable insights into the complex interactions between T_RM_ and TIME and their impact on NSCLC patient prognosis. The development of a simplified 18-gene risk score provides a practical prognostic marker for risk stratification.

## Introduction

Non-small cell lung cancer (NSCLC) accounts for ~85% of lung tumors in adults and is a leading cause of death. Various immune cell populations are present within the NSCLC tumor-immune microenvironment (TIME) (1). Among them, tissue-resident memory T (T_RM_) cells are a unique subset of T cells that permanently reside within tissues (2–5). Associated with cell surface markers including CD69, ITAG1 (CD49a), and ITGAE (CD103), T_RM_ are non-circulating memory T-cells residing in various tissues that provide an intrinsic defense system against antigens.

T_RM_ are characterized by the expression of tissue-specific homing molecules and immune exhaustion markers (6). T_RM_ cells could play a critical role in anti-tumor immune responses by either directly attacking cancer cells or indirectly promoting the recruitment of activated cytotoxic T cells to the tumor site (5, 7–9). In addition, T_RM_ cells exhibit transcriptional programs associated with tissue-resident memory and display characteristics of tumor neoantigen-specific T cells (10). Targeting T_RM_ cells for potential enhancement of immunotherapies has also been proposed (11). Studies have demonstrated that TRM cells can synergize with checkpoint inhibitors to improve anti-tumor responses. Researchers have explored strategies like adoptive T_RM_ cell transfer, inducing T_RM_ cell accumulation within tumors using cytokines like IL-33, promoting T cells to express homing receptors for tumor localization, and combining T_RM_ cell-targeting approaches with cancer vaccines (12, 13). These strategies aim to harness the localized tumor surveillance and rapid response capabilities of T_RM_ cells, potentially leading to improved efficacy of immunotherapies and durable anti-tumor immunity (13–15). However, how to evaluate the T_RM_ abundance in the TIME of NSCLC patients, and the role of T_RM_ in the TIME to affect the tumor progression and patient prognosis are still unclear. Therefore, a comprehensive understanding of how T_RM_ cells shape the NSCLC TIME and a robust gene signature for assessing T_RM_-related influence and prognosis across independent patient cohorts is much needed.

In this study, we comprehensively analyzed all publicly available single-cell datasets to extract T_RM_-related signatures representative of T_RM_ abundance in the tumor milieu. A systematic evaluation of available NSCLC single-cell and bulk RNA-seq datasets revealed the relationship of the T_RM_ with various immune cell populations in TIME and with patient prognosis. A universal 18-gene risk score derived from T_RM_ signatures across independent datasets stratified low- and high-risk NSCLC patients, distinguishing their survival. Among NSCLC patients, the T_RM_ signature has significant prognostic value for lung adenocarcinoma but not lung squamous cell carcinoma, suggesting that distinct TIME may drive different therapeutic responses in the two lung cancer subtypes.

## Materials and methods

### Data utilized in this study

The level 3 TCGA RNAseq data and clinical information involving the lung adenocarcinoma (LUAD, n=513) and lung squamous cell carcinoma (LUSC, n=501) datasets were obtained from TCGA on FireBrowse (gdac.broadinstitute.org/). TCGA MAF files for gene mutation analyses were obtained from https://gdc.cancer.gov/about-data/publications/pancanatlas. All genes in which non-silent mutations occurred were considered to be mutated. Total mutation burden was represented as the sum all non-silent mutations in a given TCGA sample. Macrophage regulation scores, leukocyte and lymphocyte infiltration scores, and IFNγ response and TGFβ response scores for TCGA-LUAD and TCGA-LUSC samples were downloaded as a **Supplementary File** (**Supplementary Table 1**) from prior work (16). The gene expression of 745 NSCLC patients and related clinical information data were collected from the GSE67639 of the open Gene Expression Omnibus (GEO) database (https://www.ncbi.nlm.nih.gov/geo/query/acc.cgi?acc=GSE67639).

### Curation of immune-related genes (IRGs)

Immune-related genes (IRGs) were obtained from **Supplementary Table 6** by Charoentong et al. (17). All genes from immune cells were collected (marker genes attributed to cancer cells were excluded) and combined into a single list of 783 IRGs genes.

### Immune cell inference

Immune infiltration scores of six immune cells were calculated using Binding Association with Sorted Expression (BASE) (18), a rank-based gene set enrichment method. Previous publications have detailed and validated immune cell infiltration using this method (18–23). BASE uses immune cell-specific weight profiles and patient gene expression data to infer immune cell infiltration for each patient and immune cell type. The BASE orders genes for a patient’s gene expression profile from high to low expression and then uses weights from each immune cell weight profile to weigh the patient’s gene expression values. BASE calculates two running sums, one representing the cumulative distribution of the patient’s weighted gene expression values (foreground function) and another representing the cumulative distribution of the patient’s complementary weighted (1-weight) gene expression values (background function). In the presence of a high amount of infiltrate from a specific immune cell type, the foreground function increases quickly, as the highly expressed genes in a patient’s profile tend to be the ones with high immune cell weights, while the background function increases slowly. The maximal absolute difference between the foreground and background functions represents the immune infiltration level and, after a normalization procedure, results in the final immune infiltration score (19, 21). Similarly, BASE was used to calculate single cell-based T_RM_ scores using T_RM_ signatures (see next section).

### Generation of T_RM_ signatures

NSCLC single cell RNA-seq datasets from human NSCLC were obtained from previous publications (10, 24). Cluster annotations were also obtained from these publications. For each NSCLC cluster, a list of marker genes was provided by identifying genes that are over-expressed in the corresponding cluster compared to all the other clusters. These cluster-specific marker gene sets were used as T_RM_ signatures. In total, 20 human T_RM_ signatures were defined, including 10 CD8+ sources and 10 generalized T_RM_ signature. The signature gene expression and the proportion of cells expressing these genes in the T_RM_8, T_RM_9, and T_RM_12 signatures were shown in the sub tables of the **Supplementary Table 2**. Given a NSCLC gene expression dataset, the BASE algorithm was used to calculate sample-specific T_RM_ scores for each signature. The T_RM_ signatures were represented as gene sets without assigning weights to the member genes. In this case, the BASE algorithm degenerated into a method like single-sample Gene Set Enrichment Analysis (ssGSEA) (25). A high T_RM_ score indicates that the corresponding T_RM_ cells are strongly infiltrated into the tumor.

### Principal component analysis

Principal component analysis (PCA) was performed using the prcomp R function. Principal component coordinates for each sample were extracted using the factoextra R package (https://github.com/kassambara/factoextra). The percentage associated with each principal component (PC) in PCA is calculated based on the amount of variance that the component accounts for in the original dataset. Mathematically, if there are n principal components with eigenvalues λ1, λ2,…, λn, the percentage for the first principal component (PC1) is calculated as:


PC1 percentage =(λ1 /(λ1 + λ2 +…+ λn))*100

This percentage represents the proportion of the total variance in the original data that is accounted for by the first principal component. The same calculation is applied to the other PCs as well. The percentage for the second PC (PC2) is calculated as:


PC2 percentage=(λ2 /(λ1 + λ2 +…+λn))*100

And so on for the remaining components. The percentages for all the PCs should sum up to 100%, as they represent the decomposition of the total variance in the dataset.

Principal component 1 (PC1) is the first principal component, and it accounts for the largest possible amount of variance in the dataset. A high PC1 percentage suggests that a significant portion of the variation in the dataset can be captured by this single component, which simplifies the interpretation of the data and allows for visualization in a lower-dimensional space. PC1 was used to represent T_RM_ infiltration.

### Estimation of stromal and immune scores

The gene expression data of LUAD and LUSC tissues in derivation population were downloaded from the Genomic Data Commons (GDC, available at: http://potal.gdc.cancer.gov/) Data Portal. The FPKM (fragments per kilobase of exon per million reads mapped) method was used to quantify gene expression. The expression matrix for estimating the stromal and immune scores was normalized by the ESTIMATE algorithm. Stromal and immune scores were calculated by performing single-sample gene set enrichment analysis. These scores formed the basis for the Estimation of STromal and Immune cells in MAlignant Tumor tissues using Expression data (ESTIMATE) score (26).

### Lasso Cox regression

The TCGA-LUAD dataset was randomly divided into a training and testing set with a 1:1 ratio. The training set was analyzed to identify potential prognostic genes and both the testing set and the entire set were used for validation. First, univariate Cox-proportional hazards regression analysis was used to evaluate the association between the overall survival and the gene expression of the gene set, which including the T_RM_ signatures’ genes and 783 IRGs. Genes with a p-value of< 0.05 based on the log-rank test were selected as candidate genes. Second, most minor absolute shrinkage and selection operator (Lasso) Cox regression analysis from the R glmnet package was employed to screen the genes most associated with overall survival in a multivariate model, which resulted in 18 genes (ABAT, AHSA1, BTN2A2, CCL20, CD109, CD200R1, CD70, CLEC17A, FST, GNG7, HSPA4, HVCN1, KIR2DL1, LTK, NEFL, RDX, and SIK1). These 18 genes composed the final risk score, which is described as follows:


$$Riskscore=\;\sum_{i=0}^{n}\beta_{i}x_{i}$$

where *βi* refers to the coefficients of each gene and $x_{i}$ represents the expression value of the gene.

### Survival analysis

For univariate and multivariate survival analyses, Cox proportional hazards models were calculated using the “coxph” function from the R “survival” package. Survival curves were visualized using Kaplan-Meier curves using the “survfit” function from the R “survival” package. Median immune cell infiltration scores were used to stratify patients into “high” and “low” groups for univariate analyses. For multivariate analyses, an infiltration score of 0 was used as separator to stratify patients into “high” and “low” groups. Differences in survival distributions in each Kaplan-Meier plot were calculated using a log-rank test using the “survdiff” function from the R “survival” package.

### Enrichment pathway analysis

The R package fgsea, version 1.26.0 (27), was used to perform GSEA with hallmark pathways from the Human Molecular Signatures Database (MSigDB) (28) to investigate which hallmark pathways were significantly (adjust P value< 0.05).

### Statistical analyses

The Spearman correlation coefficient (SCC) was reported for all correlation analyses as the assumptions underlying the Pearson correlation (i.e., normal distribution, homoscedasticity or linearity) were not met. SCC was calculated using the R function cor and significance was assessed using cor.test. The sensitivity and specificity of the diagnostic and prognostic prediction models were analyzed by the ROC curve and quantified based on the area under the ROC curve (AUC). All statistical tests were two-sided and p-values< 0.05 were considered statistically significant. All statistical analyses were performed using R software (version 4.2.0).

### Data availability

All data available in this study is publicly available. These data can be found at: gdac.broadinstitute.org/, https://gdc.cancer.gov/about-data/publications/pancanatlas. caintergator.nci.nih.gov, https://cgga.org.cn.

## Results

### Custom T_RM_ signatures representing the T_RM_ abundance in NSCLC from patient single-cell data

Associated with cell surface markers including CD69, CD49a, and CD103, T_RM_ are non-circulating memory T-cells residing in various tissues that provide an intrinsic defense system against antigens. Developed from circulating effector memory T cells in response to an antigen, T_RM_ undergo rapid proliferation upon reactivation with dual capability of both effector memory T cells and memory T cells.

**Figure 1:**
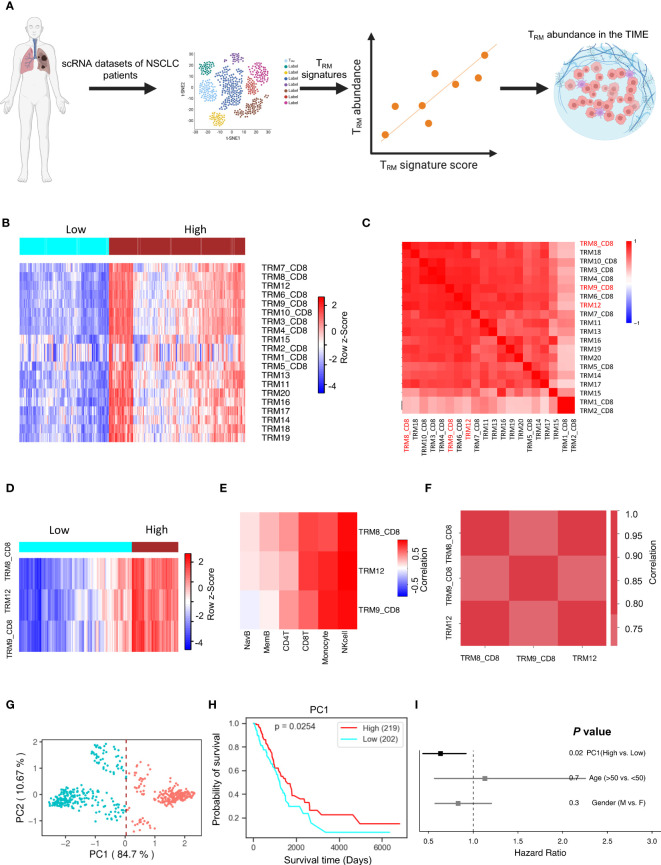
T_RM_ cell abundance is positively associated with NSCLC prognosis. The PC1 score can represent T_RM_ signatures, and it can represent the T_RM_ cell proportion in the NSCLC. **(A)** Utilizing multiple independent single-cell RNA-seq data from human NSCLC samples, we crafted 20 distinct NSCLC T_RM_ signatures reflective of T_RM_ infiltration. **(B)** The infiltration distribution of the 20 T_RM_ signatures in patients. **(C)** Heatmap of 20 T_RM_ signatures we found and the correlation of them. **(D)** The infiltration distribution of the T_RM_8, T_RM_9, and T_RM_12 signatures in patients. **(E)** The correlation of T_RM_8, T_RM_9, and T_RM_12 signatures with mainly immune cells (natural killer (NK) cells, CD8+ T cells, monocytes, memory B cells, naïve B cells, and CD4+ T cells) in patients. **(F)** The correlation of the selected T_RM_ signatures with each other. **(G)** Principal Component Analysis (PCA) on the expression of the selected T_RM_ signatures in NSCLC patients. **(H)** Kaplan-Meier plot showing the association between overall survival and the first principle component (PC1) in NSCLC. **(I)** Forest plot depicting hazard ratios of univariate Cox regression models evaluating the association between overall survival and several clinical variables. **Figure 1A** created with BioRender.com.

Binding Association with Sorted Expression (BASE), a rank-based gene set enrichment analysis method (18–23), was performed in conjunction with T_RM_ signatures. Twenty T_RM_ signatures, based on gene expression, were isolated from T_RM_ cell clusters to compile data from various independent human NSCLC single-cell RNA-sequencing (scRNA-seq) cohorts (10, 24) (**Figure 1A**). The twenty T_RM_ cell cluster gene sets are shown in **Supplementary Table 3**. This process generated a manageable form of estimated abundance data, validated in our previous studies (14, 20). Patients in TCGA-LUAD were separated into high and low T_RM_ abundance groups (**Figure 1B**). The twenty T_RM_ signatures have high correlation with each other (**Figure 1C**).

Significant signatures were chosen for further analysis, and individual signature survival analysis was conducted using Lifelines KaplanMeierFitter to visualize the results. Three T_RM_ signatures (T_RM_8, T_RM_9, and T_RM_12) are most likely correlated with NSCLC patients’ prognosis. Higher T_RM_ abundance was correlated with higher survival in the selected T_RM_ signatures (Hazard Ratio<1.0, **Supplementary Figure 1A**). When isolating the three signatures into two high and low T_RM_ infiltration groups, the outcome is depicted in **Figure 1D** showing the two groups are divided clearly and distinctly. The selected signatures had positive correlations with key immune cells like T cells, monocytes, memory B cells, naïve B cells, and especially strong correlations with natural killer (NK) cells and CD8+ T cells, in multiple independent cohorts (**Figure 1E**, **Supplementary Figure 2A**). Some kinds of these immune cells are always beneficial to patient prognosis, such as the CD8+ T cells (**Supplementary Figure 1C**). These T_RM_ signatures have very high correlations with each other in multiple independent NSCLC cohorts (**Figure 1F**, **Supplementary Figure 2B**).

### T_RM_ abundance is associated with the expression of immune checkpoint and stimulatory genes and immune regulatory pathways

Principal component analysis for dimensionality reduction captured the variance present in the highly correlated T_RM_ signatures. The first principal component (PC1) was highly correlated with all T_RM_ signatures and captured 84.7% of the variation in patients (**Figure 1G**, **Supplementary Figure 1C**), and is better associated with patient prognosis (**Figure 1H**) than conventional clinical variables including age and gender (**Figure 1I**). The PC1 could be suitable to represent the T_RM_ abundance (14). These results were then validated in an independent NSCLC dataset, GSE67639 (**Supplementary Figures 2C, D**).

**Figure 2:**
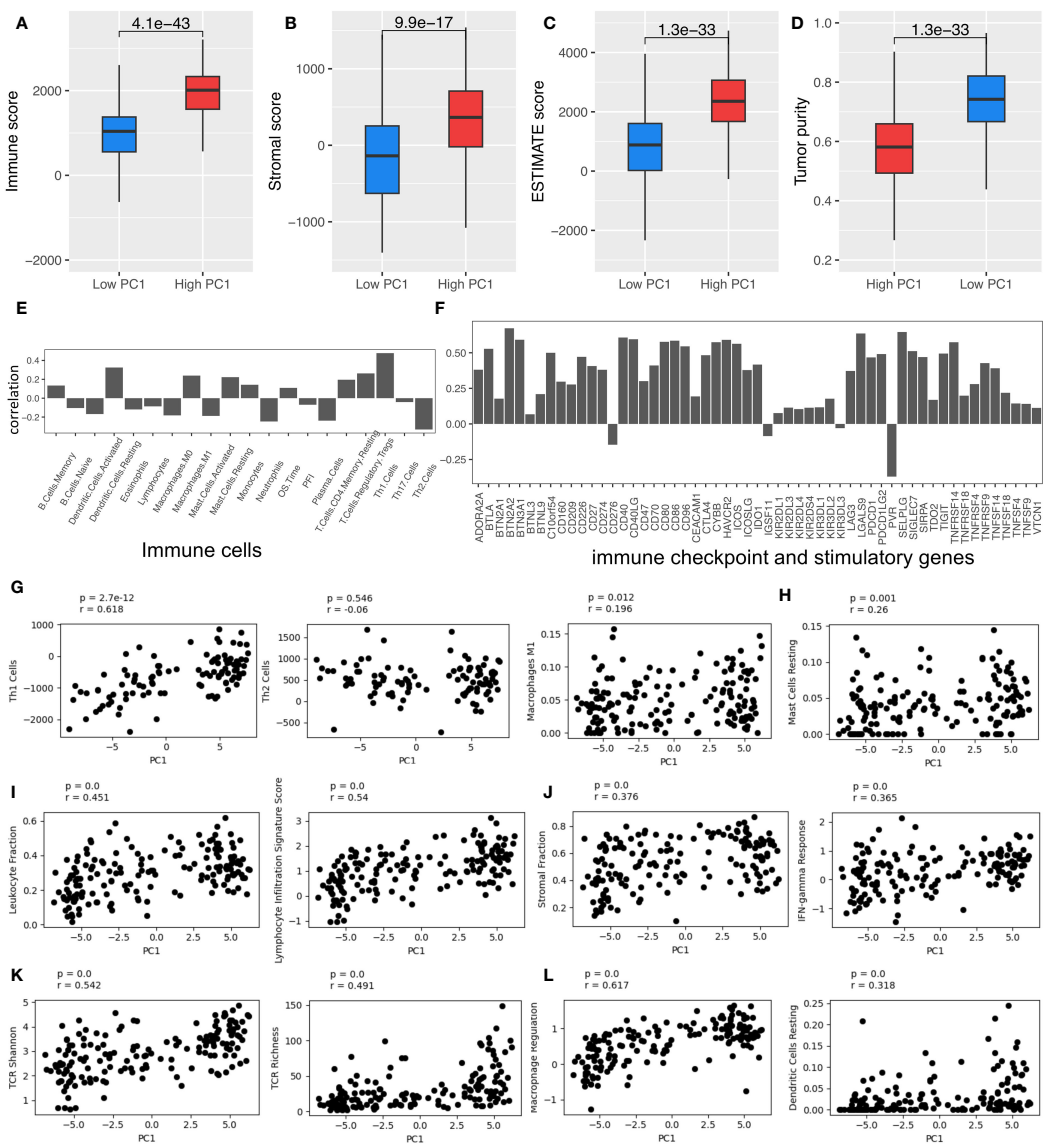
T_RM_ cell abundance is associated with the expression of immune checkpoint and stimulatory genes and immune regulatory pathways. **(A)** Immune score; **(B)** Stromal score; **(C)** Estimate score; **(D)** Tumor purity. **(E)** The Spearman correlation coefficient (SCC) between PC1 and immune cells. **(F)** SCC between PC1 and immune checkpoint and stimulatory genes expressed in NSCLC. **(G)** SCC between PC1 and Th1, Th2, and macrophages M1 cells. **(H)** SCC between PC1 and mast cells resting. **(I)** SCC between PC1 and leukocyte and lymphocyte infiltration. **(J)** SCC between PC1 and immune infiltration score. **(K)** SCC between PC1 and TCR Shannon and richness. **(L)** SCC between PC1 and macrophage regulation and dendritic cell (DC) resting. LUAD, lung adenocarcinoma.

The patients with high PC1 values also had hotter TIME, with higher immune scores, ESTIMATE scores, stromal cell scores, and lower tumor purity, which were evaluated by using the ESTIMATE algorithm (26) (**Figures 2A–D**, respectively). PC1 is positively correlated with various types of immune cells, immune checkpoint and stimulatory genes (**Supplementary Table 4**), which were identified and reported in the previous studies (29–32), respectively (**Figures 2E, F**). Furthermore, PC1 shows positive correlations with many important immune-related pathways, like leukocyte infiltration, lymphocyte infiltration, TCR richness, TCR Shannon, macrophage regulation, stromal cell infiltration, and IFN-γ response (**Figures 2G–L**). These results corroborate the derived T_RM_ signatures and their association with key immune markers, suggesting that tumors with higher T_RM_ abundance may have more active TIME.

### A prognostic tool of the risk score from an 18-gene panel for lung adenocarcinoma

**Figure 3:**
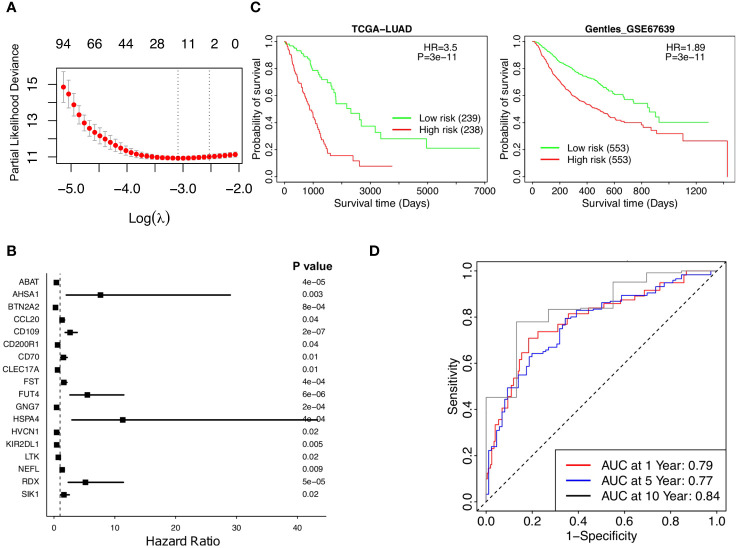
Stratified survival analysis of the 18-gene risk score model and Kaplan-Meier survival analysis for the patients in independent datasets by the 18-gene risk score model. **(A)** Development of a T_RM_ risk score for NSCLC patients by Lasso Cox regression analysis. **(B)** The forest plot of the 18 genes in the risk model. **(C)** Patients in the TCGA-LUAD and GSE67639 cohorts. **(D)** Time-dependence of NSCLC in 1, 5, and 10 years, respectively.

Abundance data based on the T_RM_ signatures is formed from a large number of genes composing a final infiltration score. The Lasso Cox regression model identified 18 genes significantly associated with patient survival (**Figures 3A, B**).

A risk score based on these 18 genes was calculated in the form of $riskscore=\;\sum_{i=0}^{n}\beta_{i}x_{i}$ , where corresponds to the weight of each gene and $x_{i}$ corresponded to the expression of that gene in the patients’ cancer tissues. The weights of the 18 genes are shown in **Supplementary Table 5**. A higher weight represented more prognostic significance for that gene. Weighted gene expressions were coalesced into a risk score for each patient (**Figure 3A**). Each of the 18 genes is correlated with patient survival (**Figure 3B**), the overall risk score provides a much significant p-value as the low-risk patients had much higher survival than the high-risk patients according to Kaplan-Meier analysis of patients in TCGA-LUAD (p value =3e-11, HR =3.5, **Figure 3C**, left). The prognostic significance of the risk score was further supported by the independent dataset, GSE67639, where survival once again significantly favors the low-risk patients (**Figure 3C**, right). More low-risk patients have a high T_RM_ abundance and have survived (**Supplementary Figures 3A, B**). The predicted AUC reached 0.79, 0.77, and 0.84 in 1, 5, and 10 years, respectively (**Figure 3D**). Taken together, lower risk patients had significantly higher survival; the 18-gene risk score is a strong independent prognostic risk factor for patients with NSCLC (**Figure 3**).

### Performance of the risk model with respect to clinicopathological factors

**Figure 4:**
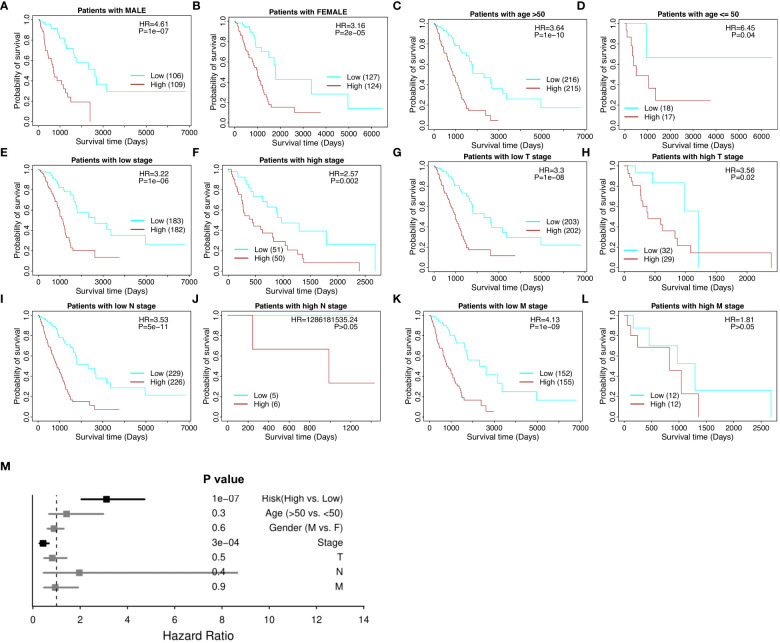
Stratified survival analysis of the 18-gene risk score model in clinicopathological factors. **(A)** The risk model in male patients. **(B)** The risk model in female patients. **(C)** The risk model in the elderly (age > 50). **(D)** The risk model in the young (age ≤ 50). **(E)** The risk model in low tumor stage patients. **(F)** The risk model in high tumor stage patients. For the TNM cancer staging system, TNM stands for Tumor, Nodes, and Metastasis. T is assigned based on the extent of involvement at the primary tumor site, N for the extent of involvement in regional lymph nodes, and M for distant spread. **(G)** The risk model in low T stage patients. **(H)** The risk model in high T stage patients. **(I)** The risk model in low N stage patients. **(J)** The risk model in high N stage patients. **(K)** The risk model in low M stage patients. **(L)** The risk model in high M stage patients. **(M)** Multivariate independent prognosis analysis in NSCLC cohort.

The risk model proves effective with respect to patient cohorts separated into male patients and female patients (**Figures 4A, B**), age over and under 50 (**Figures 4C, D**), high and low tumor stage progression (**Figures 4E, F**), and TNM cancer staging (T stages in **Figures 4G** and **H**, N stages in **Figures 4I** and **J**, and M stages in **Figures 4K** and **L**, respectively). T is assigned based on the extent of involvement at the primary tumor site, N for the extent of involvement in regional lymph nodes, and M for distant spread. Furthermore, in the multivariate model, the risk score maintained its significance and significantly outperformed the other clinical variables, including gender, age, stage, and stages T, N, and M (**Figure 4M**). Therefore, the 18-gene risk model remains an effective prognostic tool when weighed against current clinicopathological factors for patient prognosis.

### The risk score correlated with immune cell infiltration and regulatory pathways

**Figure 5:**
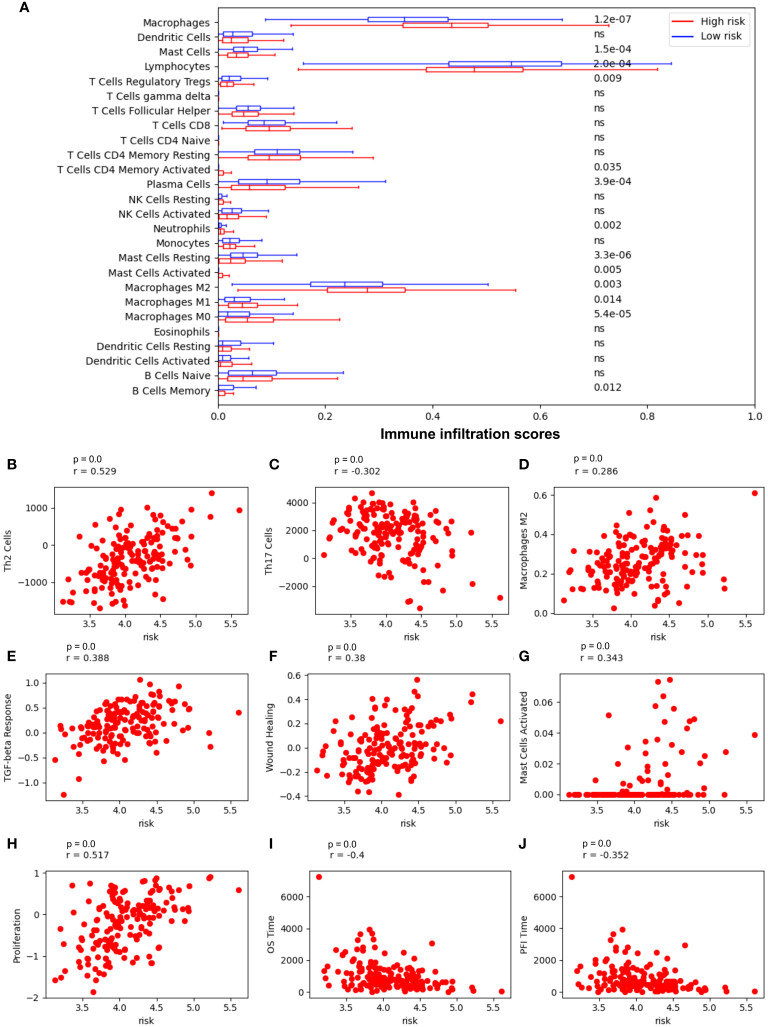
Risk model is most associated with immune cells in NSCLC. **(A)** Immune cell infiltration in low-risk vs high-risk patients. **(B)** The Spearman correlation coefficient (SCC) between risk score and Th2 cells; **(C)** SCC between risk score and Th17 cells; **(D)** SCC between risk score and macrophages M2 cells; **(E)** SCC between risk score and the transforming growth factor beta (TGFβ) response; **(F)** SCC between risk score and wound Healing; **(G)** SCC between risk score and mast cells activated; **(H)** SCC between risk score and tumor proliferation; **(I)** SCC between risk score and overall survival (OS) time; **(J)** SCC between risk score and Progression-Free Interval (PFI) time.

The risk model correlated with immune cell infiltration and relevant immune pathways (**Figure 5**). Low-risk patients have markedly higher infiltration of lymphocytes, mast cells, and memory B cells (p value = 2.0-e4, 1.5e-4, 0.012, respectively), whereas high-risk patients have higher infiltration of neutrophil and macrophages M2 (p value = 0.002 and p value = 0.003, respectively), suggesting more beneficial TIME characteristics for patients with low-risk scores (**Figure 5A**). Notably, low-risk scores correspond to a high number of Th17 cells, but a lower number of Th2 cells and macrophages M2 infiltration (**Figures 5B–D**, respectively), whereas high-risk scores are positively correlated with TGF-beta response, wound healing, mast cell activation, and tumor proliferation (**Figures 5E-H**, respectively) and negatively correlated with patients’ overall survival time and progression-free interval (PF1) time (**Figures 5I, J**, respectively).

### The patients with low risk scores and high T_RM_ abundance exhibit enrichment for active immune pathways

**Figure 6:**
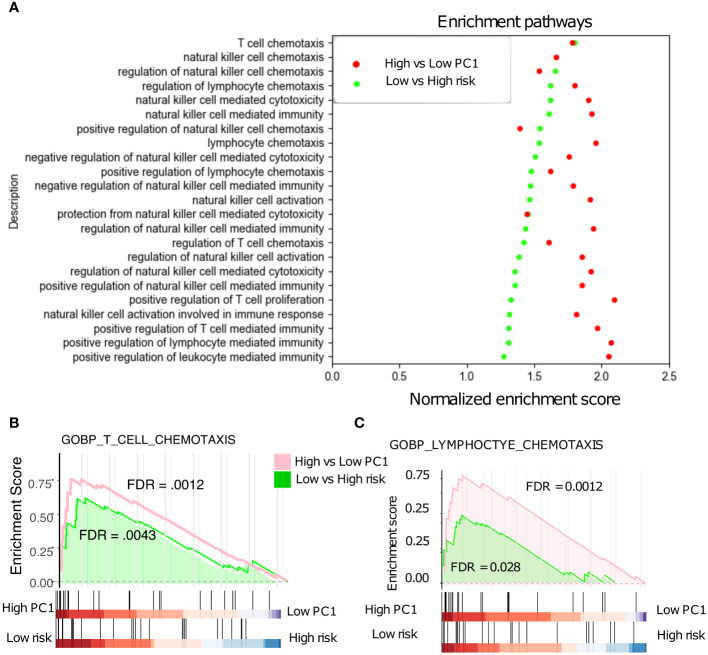
Gene Set Enrichment Analysis (GSEA). **(A)** Low-risk patients with significant up-regulated T cell, NK cell, and Lymphocyte -related pathways in the NSCLC. **(B)** T cell chemotaxis pathways are significantly up-regulated in low- vs high- risk patients. **(C)** Lymphocyte chemotaxis pathways are also significantly up-regulated in low- vs high- risk patients.

The 18-gene risk score was further evaluated against various Gene Ontology biological processes (GOBPs) by the gene set enrichment analysis (GSEA) and the associated Molecular Signatures Database (MSigDB) (28). The NSCLC patients with low risk scores and high T_RM_ abundance are associated with the upregulation of various T cells, natural killer cells, and lymphocytes related immune pathways (**Figure 6A**). Especially, these patients showed a similar correlation with pathways such as T cell and lymphocyte chemotaxis (**Figures 6B, C**), affirming the connection between PC1, risk, and patient prognosis.

### Differential T_RM_ abundance and its prognostic value in lung adenocarcinoma compared with lung squamous cell carcinoma

**Figure 7:**
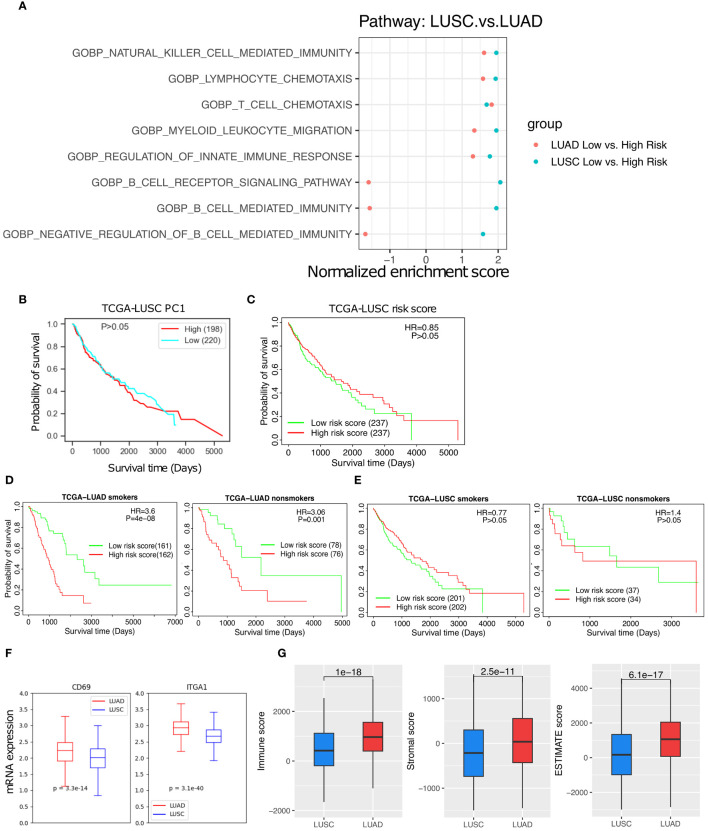
The T_RM_ abundance difference between the LUAD and LUSC. **(A)** The T cell, NK cell, and immune response related pathways are significantly up-regulated in low- vs high- risk of LUSC and LUAD patients. **(B)** High PC1 vs low PC1 LUSC patient survival. **(C)** High risk vs low risk in LUSC patient survival. **(D)** High risk vs low risk in LUAD smoker patients and non-smoker patients. **(E)** High risk vs low risk in LUSC smoker patients and LUSC non-smoker patients. **(F)** T_RM_ marker genes expression difference between the LUAD and LUSC patients. **(G)** Immune score, Stromal score, and Estimate score, respectively.

Lung adenocarcinoma (LUAD) and lung squamous cell carcinoma (LUSC) are two main subtypes of NSCLC. The T_RM_ abundance are positively correlated with various anti-tumor immune cells in both subtypes, but its correlation with B cell mediated immunity pathways are noticeably different between LUAD and LUSC (**Figure 7A**, **Supplementary Figure 4**). In contrast to LUAD (**Figure 3C**), neither the T_RM_ PC1 nor the 18-gene risk score was able to distinguish any difference in survival among LUSC patients (**Figures 7B, C**). Furthermore, the T_RM_ score distinguished prognostic differences for both smoker or non-smoker populations respectively in LUAC (**Figure 7D**) but not in LUSC (**Figure 7E**). The T_RM_ marker genes expression seem to be much lower in LUSC than in LUAD patients (**Figure 7F**). The immune score, stromal score, and ESTIMATE score (26), indicators of how hot or cold the TIME is, are also lower in LUCS than LUAD (p = 1e-18, 2.5e-11, and 6.1e-17, respectively, **Figures 7G**).

## Discussion

T_RM_ are a specialized population of T cells that reside in peripheral tissues, especially in the lung and skin (33, 34). Our comprehensive meta-analysis generated signatures of T_RM_ abundance in NSCLC patients using available single-cell RNA-sequencing (scRNA-seq) data. We provide evidence that T_RM_ signatures are indicative of prognosis and immune responses in NSCLC. A higher T_RM_ abundance was correlated with higher survival and better prognostic outcome in NSCLC patients. Furthermore, the T_RM_ signatures demonstrated strong correlations with the presence of immune cells such as the CD8+ T cells and NK cell in the TIME, which are known to impact patient prognosis (35). Higher T_RM_ abundance in the TIME is associated with higher degree of immune infiltration and ‘hotter’ TIME (**Figure 3**). Infiltration by leukocytes, lymphocytes, stromal, and DC cells are positively correlated with T_RM_ whereas Th2 cells and M2 macrophages are negatively correlated with T_RM_ in NSCLC patients (**Figure 2**).

An 18-gene risk score for lung adenocarcinoma prognosis was developed, which are associated with T cell functions and demonstrate significant associations with patient survival (36–49). The risk model has better prognostic associations than various clinicopathological factors, such as the gender, age, and stages. Similar patterns and immune regulation results were observed in low- vs high- risk patients and high- vs low- T_RM_ abundance patients (**Figure 6**). The roles of most genes in activation, metabolism, regulation, inflammation of T cells, and other immune functions, have been solidly established in literature (36–49). For example, ABAT-dependent mitochondrial anaplerosis is critical for T cell-mediated inflammation (36), AHSA1 is involved in the T-cell activation pathway and related pathways (37), and BTN2A2 can inhibit the proliferation of CD4 and CD8 T-cells activated by anti-CD3 antibodies, T-cell metabolism, IL2, and IFN-γ secretion (42). CCL20 is responsible for the chemotaxis of dendritic cells (DC), effector/memory T-cells, and B-cells (43). CD109 could activate T cells (44). CD200R1 might play an important role in immunoregulation, which suppress T cell function and inflammation through DC apoptosis and polarization of macrophages toward M2 subtype (45). Tregs stably-expressing CD70 will lost their regulatory functions but activates cytolytic T cells instead (46). FUT4 was reported to be involved in PD-1-related immunosuppression and could affect operable lung adenocarcinoma patient survival. The overexpression of HVCN1 on CD8+ T cells could enhance adoptive T cell transfer immunotherapy (47), and KIR2DL1 plays a unique opposite function in CD4^+^ T cells when interacting with SHP-2 and/or SHP-1 proteins (48). The other 8 genes, CLEC17A, FST, GNG7 (49), HSPA4 (38), LTK (39), NEFL, RDX (40), and SIK1 (41), have been associated with general immune or tumor suppression pathways (38–41, 49). The correlation between immune response and some of the genes in the 18-gene list like CCL20, CD109 and CD200R1 were also identified in other studies (50–52). Given the significant correlations with T_RM_ observed in the current multi-omics study, those genes related to tumor immunity and T cell interaction that have not been well-studied will be applied for future biological investigation. We compared the results of PC1, risk model, and the risk model ROC using T_RM8,9,12_ and T_RM9,12_, respectively. There are no significant differences between them. The performance of the risk model based on T_RM8,9,12_ (AUC = 0.79, 0.77, and 0.84 in 1, 5, and 10 years, respectively) is better than T_RM9,12_ (AUC = 0.77, 0.73, and 0.74 in 1, 5, and 10 years, respectively), for the NSCLC patient prognosis (**Figure 3**, **Supplementary Figure 5**).

Surprisingly, the T_RM_ risk model was strongly predictive of prognosis and survival in lung adenocarcinoma but not in lung squamous cell carcinoma at all, although both are categorized as NSCLC. The potential cause could be that the TIME of lung squamous cell carcinoma has lower T_RM_ abundance and a colder TIME than lung adenocarcinoma as our analytical scores indicate. Understanding the nuanced roles of T_RM_ in TIME in lung cancer subtypes may aid the efficacy of emerging treatment approaches (53–55).

Altogether, the study highlights the importance of T_RM_ in the TIME and their potential as a prognostic tool for NSCLC. In addition to cancer, our analytical method may also be applied to understanding the potential role of T_RM_ in other immune-related diseases, such as rheumatoid arthritis, systemic lupus erythematosus, type 1 diabetes, multiple sclerosis, psoriasis, inflammatory bowel disease, autoimmune thyroid disease, etc. (56–58).

## Data availability statement

The datasets presented in this study can be found in online repositories. The names of the repository/repositories and accession number(s) can be found in the article/**Supplementary material**.

## Author contributions

CJ: Writing – review & editing, Writing – original draft, Supervision, Project administration, Methodology. AS: Writing – review & editing, Writing – original draft, Visualization, Formal analysis, Data curation. AG: Writing – review & editing, Investigation. C-CC: Writing – review & editing, Investigation. DL: Writing – review & editing. CC: Writing – review & editing, Investigation, Visualization. ZW: Writing – review & editing, Conceptualization, Investigation. CQ: Writing – review & editing, Conceptualization, Validation. YZ: Writing – review & editing, Investigation. JM: Writing – review & editing, Investigation.
