# Supplementary material for: A comprehensive meta-analysis of tissue resident memory T cells and their roles in shaping immune microenvironment and patient prognosis in non-small cell lung cancer
Source: Front Immunol. 2024 Jul 8;15:1416751. doi: 10.3389/fimmu.2024.1416751 (PMC11260734; doi:10.3389/fimmu.2024.1416751)
Supplement: Supplementary file 1 [file DataSheet_1.docx]

**Supplementary Figures**

**Supplementary Figure 1.**

**
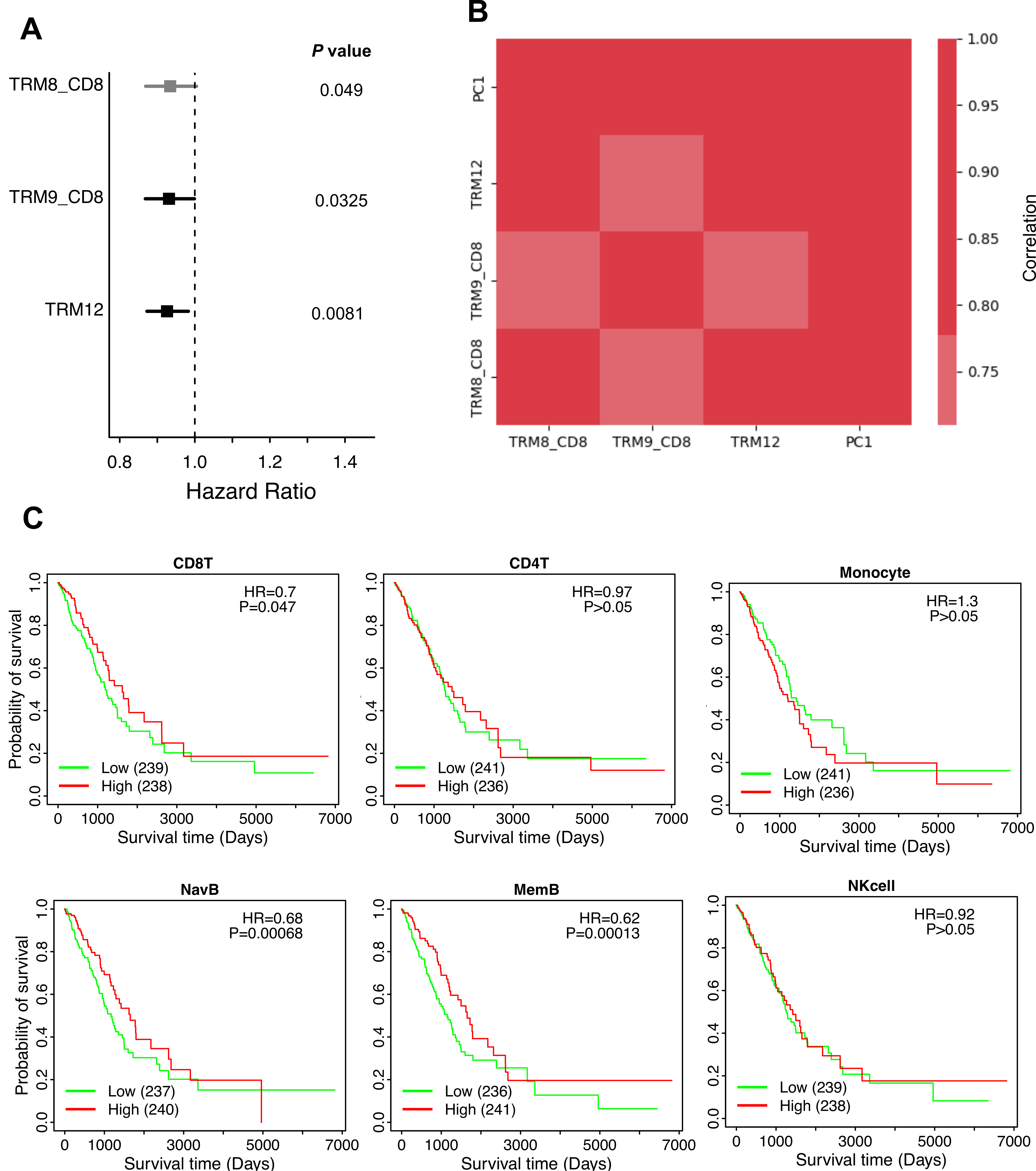
**

**Supplementary Figure 1. The T_RM_ signatures selection in NSCLC.** (A) The forest plot of the T_RM_8, T_RM_9, and T_RM_12 signatures in NSCLC patient prognosis. (B) The correlation of the T_RM_8, T_RM_9, and T_RM_12 signatures and PC1 with each other. (D) Survival analysis for the main immune cells infiltration, such as the CD8+ T cells, CD4+ T cells, NK cells, naïve B cells, memory B cells, and monocyte cells.

**Supplementary Figure 2.**

**
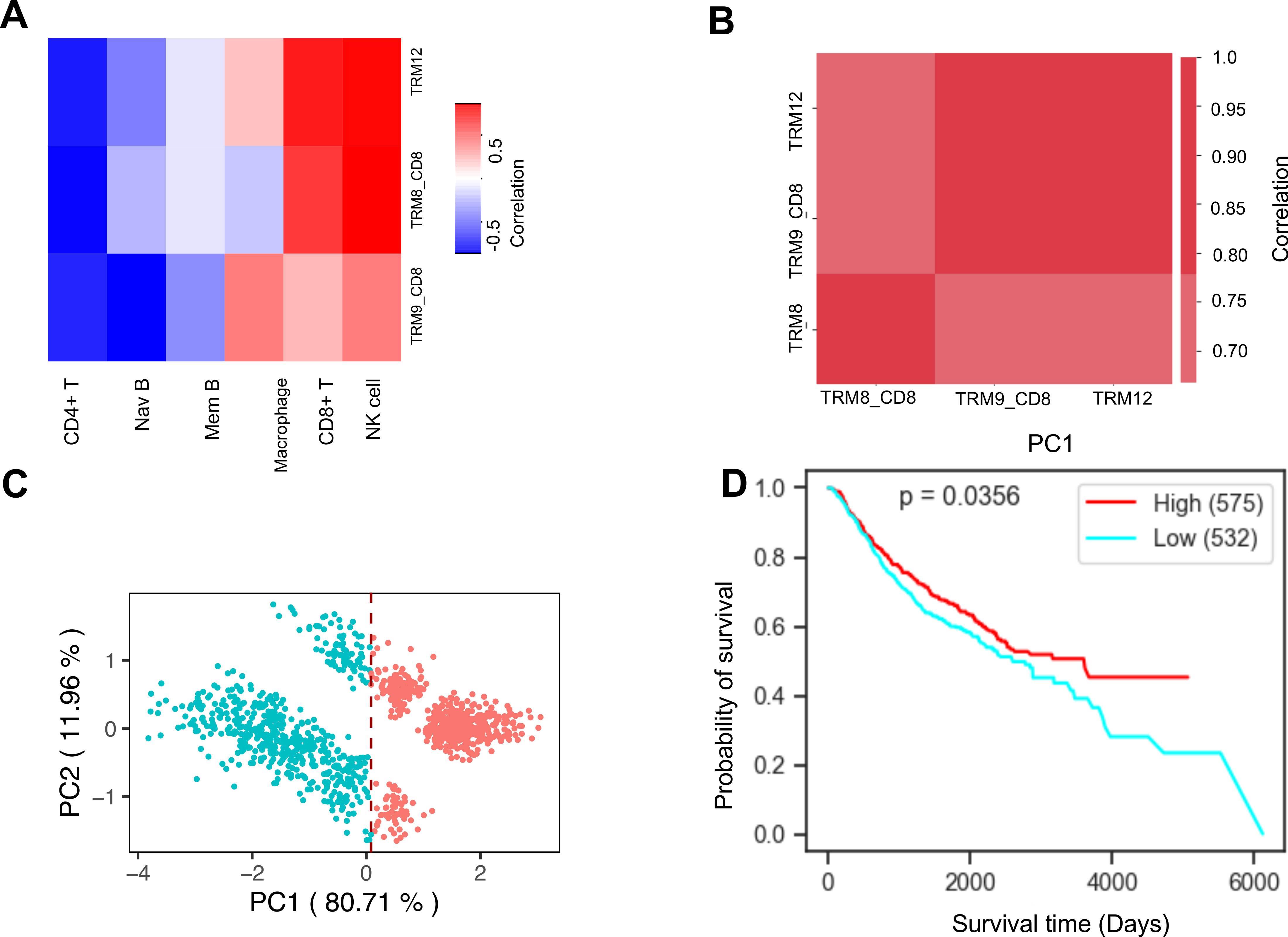
**

**Supplementary Figure 2. GSE67639. T_RM_ cell abundance is positively associated with NSCLC prognosis. The PC1 score can represent the 3 T_RM_ signatures and the T_RM_ cell proportion in NSCLC.** (A) The correlation of T_RM_ signatures with immune infiltration in NSCLC. (B) The correlation of the T_RM_ signatures with each other. (C) Principal Component Analysis (PCA) on the expression of the selected T_RM_ signatures in NSCLC patients. (D) Kaplan-Meier plot showing the association between overall survival and PC1 in NSCLC.

**Supplementary Figure 3.**

**
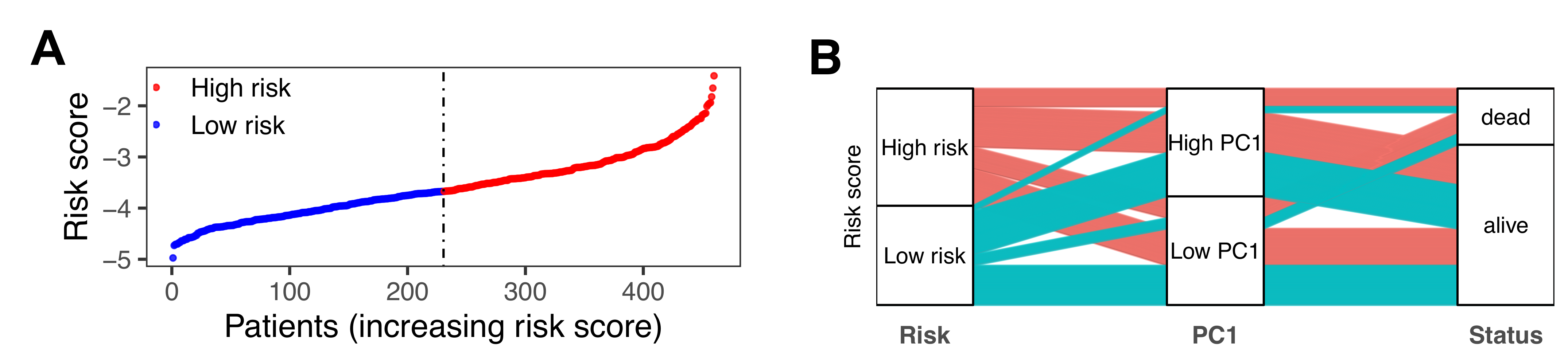
**

**Supplementary Figure 3. Survival analysis of the 18-gene risk score model in NSCLC patients.** (A) The risk score distribution in high- and low-risk groups. (B) The relationships among the risk score, T_RM_ abundance, and survival status in NSCLC patients.

**Supplementary Figure 4**

**
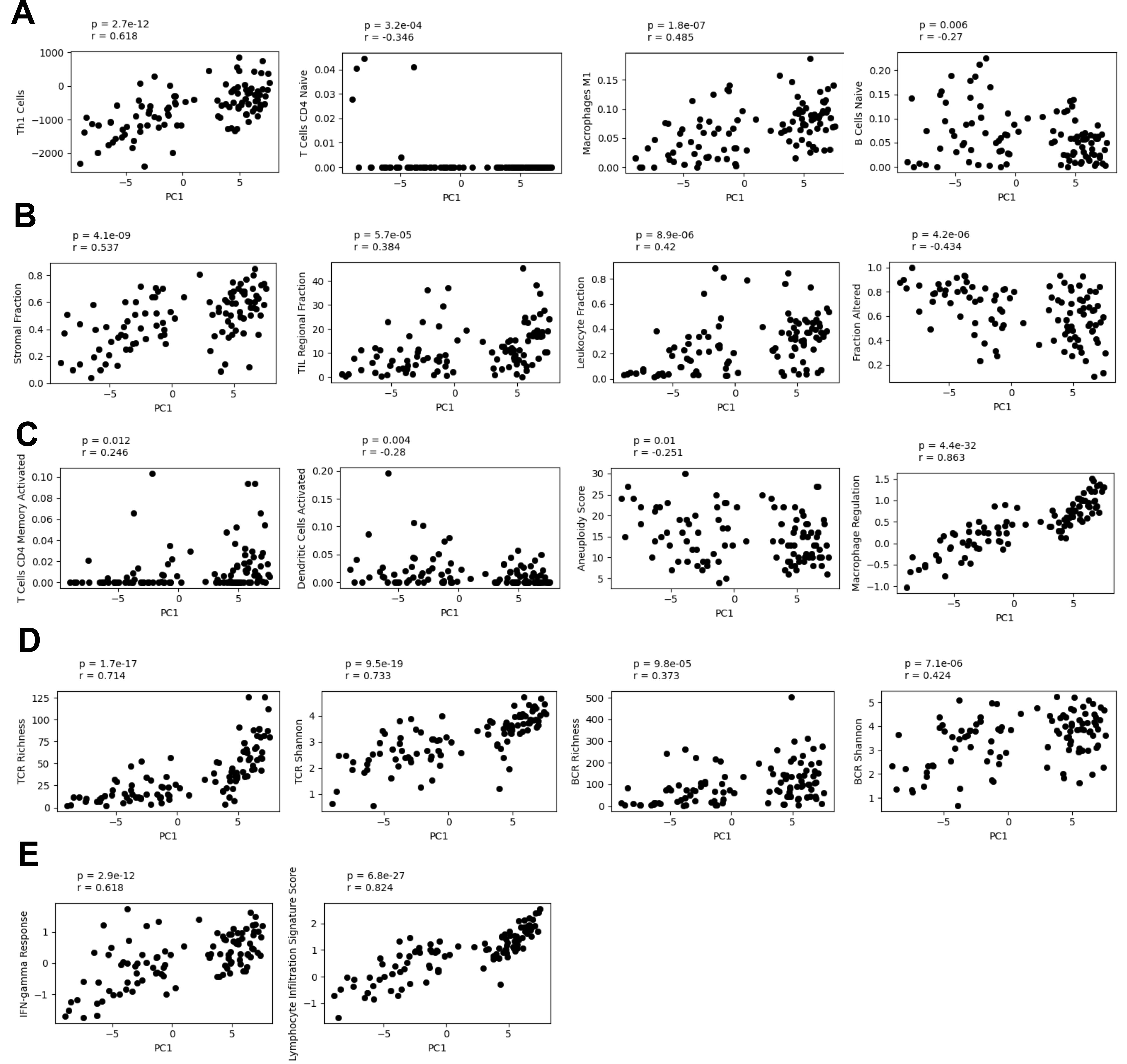
**

**Supplementary Figure 4. LUSC. T_RM_ cell abundance is associated with the expression of immune checkpoint genes and immune regulatory pathways.** (A) The Spearman correlation coefficient (SCC) between PC1 and the important immune cells. (B) SCC between PC1 and stromal cells, TIL, and Leukocyte infiltration. (C) SCC between PC1 and immune cells activated and regulation pathways. (D) SCC between PC1 and T cell related immune pathways. (E) SCC between PC1 and IFN-γ response and lymphocyte infiltration.

**Supplementary Figure 5**

**
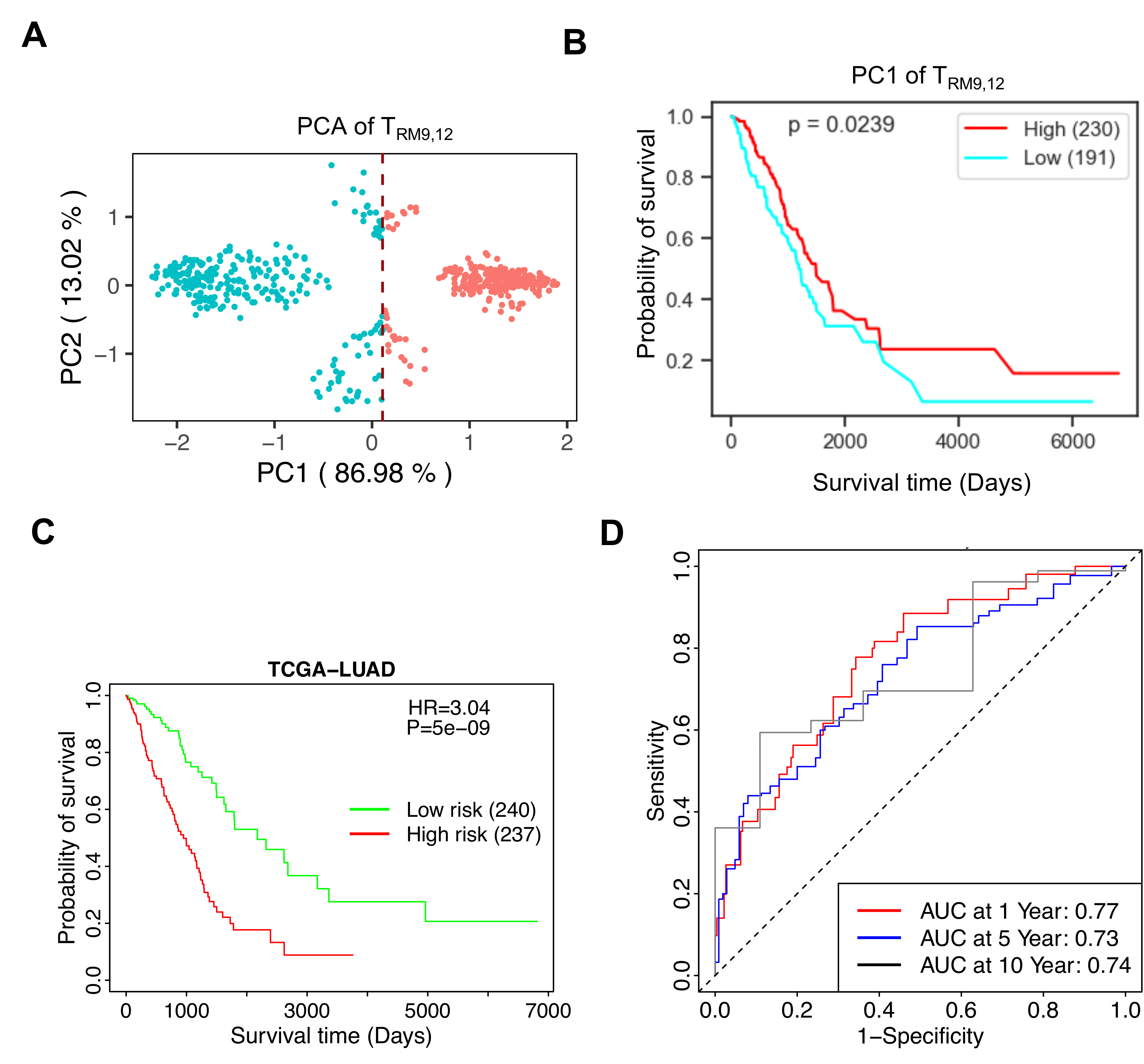
**

**Supplementary Figure 5. Survival analysis of PC1 and risk model based on the T_RM_9 and T_RM_12 (T_RM9,12_).** (A) PCA on the expression of the selected T_RM_ signatures in NSCLC patients, T_RM9,12_. (B) Kaplan-Meier plot showing the association between overall survival and the PC1 in NSCLC, T_RM9,12_. (C) T_RM9,12_. Patients in the TCGA-LUAD dataset. (D) T_RM9,12_. Time-dependence of NSCLC in 1, 5, and 10 years, respectively.
